# COVID-19 alters the relationship between relational mobility and helping behavior

**DOI:** 10.3389/fpsyg.2022.1005235

**Published:** 2022-10-05

**Authors:** Xiaoxiao Zhang, Xian Zhao, Gengnan Liao, Yuanlin Huang, Xuan Fang

**Affiliations:** ^1^Department of Psychology, Shenzhen University, Shenzhen, China; ^2^Department of Marketing, Northwestern University, Chicago, IL, United States

**Keywords:** relational mobility, helping, COVID-19, socioecology, phone call study

## Abstract

To determine if helping behaviors are affected by socioecological variables such as relational mobility and the COVID-19 pandemic, we investigated the impact of relational mobility on helping behaviors before (Study 1) and during (Study 2) COVID-19 in China *via* two experiments. In Study 1, we manipulated participants’ relational mobility and found that a greater proportion of participants in the high relational mobility condition signed up for another psychological experiment, relative to the low relational mobility condition. In Study 2, the manipulation of relational mobility was embedded in a phone interview, and we found that a high relational mobility condition caused fewer signups for a COVID-19 support program relative to a low relational mobility condition. These results extend our understanding of the meaning of relational mobility under different ecological contexts.

## Introduction

The COVID-19 pandemic substantially changes our everyday life and behaviors (Alon et al., 2020; Van Bavel et al., 2020). Helping behavior is valuable and essential when such global epidemic is prevalent. Literatures have shown some unique predictors of helping behavior (e.g., self-transcendence; Politi et al., 2021) in the period of the COVID-19 pandemic. Researchers have also found that helping behavior promoted individual’s well-being to cope with the COVID-19 (Espinosa et al., 2022). In this study, we aimed to provide findings to show how people engage in opposite patterns of helping behaviors before and during the COVID-19 pandemic. When and why do people help others? Researchers investigated this topic from individual (e.g., Vos and van der Zee, 2011), interpersonal (e.g., Darley and Latane, 1968), and intergroup (e.g., Sparkman and Hamer, 2020) perspectives. Some classic psychology literature (e.g., Latané et al., 1970) postulates that helping depends on how potential helpers cognitively process the immediate situation. However, helping is also relational, with individuals offering efforts and resources to benefit others. We propose that the broader relational systems that individuals inhabit may explain the variation in helping behaviors. Generally, we argue that a relational environment with more relational opportunities may facilitate helping behaviors and a relational environment with more risks may decrease helping behaviors.

### Relational mobility and helping

Relational mobility, a socioecological concept, is the perception of freedom and opportunities to acquire new, maintain current, and terminate old relationships in social environments (Schug et al., 2010; Thomson et al., 2018). In theory, high mobility settings promote relationality as voluntary associations (Esiaka et al., 2020). This resonates with the equality matching model (Fiske, 1991) that relationships are constructed on a reciprocity principle (such as college roommates), and the market pricing model in which relationships are commonly scaled by monetary exchanges (such as buyer–seller). High relational mobility thus creates a free market of relationships where connections are mutually selected and voluntarily built. Individuals may have to show affable interpersonal characteristics and employ various strategies to stand out in mutual selection, attract valuable others, and be selected over competitors (Yuki and Schug, 2020). Their strategies may include enhanced generosity (Barclay, 2016) and self-disclosure (Schug et al., 2010) with or without the explicit goal of relationship building. Therefore, a high relational mobility environment may prompt helping in general.

### The COVID-19 alters the relationship between relational mobility and helping

Although high relational mobility creates opportunities for interpersonal connections, it may also signal danger if interpersonal connections bring risks. The pathogen prevalence theory argues that a high prevalence of communicable diseases made it dangerous to contact strangers (Fincher et al., 2008). During the COVID-19 pandemic, high relational mobility increases not only opportunities to meet strangers but also infection risks due to the fluidity of relationships (Salvador et al., 2020). To cope with infectious diseases, human beings have developed a behavioral immune system (Schaller and Murray, 2011) whereby we engage in behaviors – such as avoiding strangers – to prevent contact with pathogens (Fincher et al., 2008). Therefore, during a pandemic a high relational mobility setting may reduce our willingness to contact strangers. However, contact is a prerequisite for many helping situations. Hence, we hypothesized that higher relational mobility would enhance helping in general, but prompt lower helping during the COVID-19 pandemic.

### Overview of the present research

We conducted two experiments in China to investigate the effect of relational mobility on helping before and during the COVID-19 pandemic. Study 1 was conducted from October to December 2019, before the outbreak. Study 2 was conducted from May to September 2020, after the outbreak but still during the pandemic.

## Study 1: Relational mobility and helping before COVID-19

### Method

#### Participants

As this is the first study about this topic, we used the effect size of residential mobility on helping an outgroup member (*d* = 0.67) in a previous study (Li et al., 2019) as a proxy to conduct a power analysis based on the design of the current study. Results indicated a minimum sample size of 72 to achieve a power of 80% (Faul et al., 2007). We recruited 171 college students from Shenzhen University of China (71.20% female; *M*_age_ = 20.24, *SD*_age_ = 1.73) and assigned them randomly either a high (*n* = 82) or low (*n* = 89) relational mobility condition.

#### Measures

##### Relational mobility manipulation

We used a paragraph that contained relational mobility manipulation adapted from Li et al. (2016). In the high mobility condition, participants are informed that their surrounding environments offered many opportunities to meet new people and choose connections. However, they also face situations where they may be selected or risk being abandoned by others. In the low mobility condition, participants are told that their interpersonal relationships are relatively stable, reflecting good mutual understanding, but their surrounding environment provides limited opportunities to meet new people.

##### Manipulation check

We used a 12-item relational mobility scale developed by Thomson et al. (2018) to assess participants’ perceptions of relational mobility in the environment. A sample item is “*In most circumstances*, *it is easy for people to make new acquaintances*.” Participants were asked to indicate their agreement with each item on a 7-point Likert scale, ranging from 1 (totally disagree) to 7 (totally agree). Higher scores indicate a higher level of perceived relational mobility (α = 0.79).

##### Helping behavior

**T**he helping behavior was observed by whether the participants left their contact information (WeChat ID or email address) to sign up for free participation in another experiment^1^ (Van Lange et al., 2011). If the participants left their contact information to sign up for another experiment, we recorded them as offering help, otherwise, we recorded them as not offering help.

#### Procedure

Participants were recruited in the campus and were send a survey link. For the manipulation of relational mobility, they had to read the paragraph concerning relational mobility manipulation. Thereafter, participants had to document how their interpersonal relationships were similar with the manipulated context and provide three examples of their interpersonal relationship patterns that supported the manipulated context. Following the above, participants completed the relational mobility scale as part of the manipulation check. At the end of the questionnaire, we asked participants if they were willing to help us for another experiment without any payment which would take at least an hour in the lab. Should they be willing, they had to provide their contact information to the experimenter.

### Results

#### Manipulation check

We conducted an independent sample *t-test* to examine the effects of manipulation on the check questions. Results showed that participants in the high relational mobility condition reported a greater average score on the relational mobility scale (*M* = 4.44, *SD* = 0.63) than participants in the low mobility condition (*M* = 3.93, *SD* = 0.74), *t*(169) = 4.84, *p*<0.001, *d* = 0.62, 95% CI [0.30, 0.72]. This indicates that the manipulation was successful.

#### Effect of relational mobility on helping

We conducted a chi-square test to examine the effect of relational mobility manipulation on sign up behavior. The results showed that 32.93% (*N* = 27) of the participants in the high mobility condition left their contact information compared to 14.61% (*N* = 13) in the low mobility condition (odds ratio = 2.87, *χ^2^* = 7.99, *df* = 1, value = 0.21, *95%CI* [0.08, 0.34], *p* = 0.005). This supported our hypothesis that participants in the high relational mobility condition were more likely to helping than those in the low condition before COVID-19.

## Study 2: Relational mobility and helping during COVID-19

Study 2 was conducted while Chinese universities were in lock-down. We could not use the same way as in Study 1 to recruit participants on campus. Instead, experiments were conducted *via* phone calls.

### Method

#### Participants

We used the effect size of Study 1 (odds ratio = 2.87) as a proxy to conduct a power analysis based on the design of the current study. The results only indicated a minimum sample size of 25 to achieve a power of 95%. However, following recommendations from the field to recruit larger samples, we contacted 228 Shenzhen University students of whom 118 (female = 68.6%; male = 31.4%) agreed to participate.^2^ Participants were randomly assigned to either a high (*n* = 59) or low (*n* = 59) relational mobility condition.

#### Measures

##### Manipulation of relational mobility

The manipulation of relational mobility was similar with Study 1.

##### Helping behavior

In order to conceal the true purpose of the study and be more in line with the epidemic context, helping behavior was assessed by a sign-up behavior for a helping group to provide support for people who were isolated and suspected of being infected by COVID-19.

#### Procedure

We trained two undergraduate psychology students^3^ (one male and one female) as interviewers. To avoid direct physical contact and ensure the safety of the participants and the interviewers, students were interviewed telephonically. The interviewers asked the students if they could interview them briefly about the current living environment.^4^ The relational-mobility-manipulation material was similar with Study 1, but it was embedded in interviews this time. Experimenters read the manipulated information to participants before asking them to a) describe what interpersonal relationships are like in high or low relational mobility conditions, and b) provide three examples of the two manipulated conditions.

After the session, we assessed the participants’ helping behavior by asking if they would be willing to participate in another helping group activity which involved providing assistance to students during the pandemic, for example, delivering living supplies to isolated students. Those who agreed, were told to send their telephone numbers to the helping group for further contact. The recruitment of participants naturally ended when the campuses reopened.

### Results

We conducted a chi-square test to examine the effect of relational mobility manipulation on helping. The results for Study 2 showed that 72.88% (*N* = 43) and 88.14% (*N* = 52) of the participants in the high and low relational mobility conditions (odds ratio = 0.36, *χ^2^* = 4.37, *df* = 1, value = 0.19, 95%CI [0.02, 0.34], *p* = 0.036), respectively, left their contact information. The findings showed a different relationship between relational mobility and helping behavior after the breakout of the COVID-19.

## Discussion

The findings showed different patterns of the casual relationship between relational mobility and helping, before (Study 1) and during (Study 2) the COVID-19. Before the pandemic, our findings were generally consistent with Li et al. (2019) study which found that higher relational mobility prompted more helping behavior toward a stranger. However, the pandemic seemed to reverse this effect.

Our findings provide a new perspective for understanding helping behaviors. From a socioecological perspective, relational environment does not only entail a mobile-stable dimension (i.e., relational mobility, Yuki and Schug, 2012), but also includes a safe-danger dimension (i.e., the risk of epidemic infection, Salvador et al., 2020). During the COVID-19 pandemic, high relational mobility also indicates a physically dangerous environment. Hence, to avoid the risk caused by the high mobile relationships, people are less willing to helping during the COVID-19. Notably, our findings echo the recent discussion of globalization in psychology (Prilleltensky, 2012) or de-globalization (i.e., Witt, 2019). The COVID-19 may decrease people’s acceptance of globalization and promote the progress of the de-globalization.

This research also has limitations. First, the study did not statistically test a moderation model in which the presence of COVID-19 moderates the effect of relational mobility on helping. Future studies could design a 2 by 2 experiment and test the hypothesis more rigorous. Second, although we proposed that the perceived risk of being infected is the mechanism between relational mobility and helping, it should be measured in future studies. Of course, there may be other explanations for this result, for example, expected reciprocity (Simpson and Willer, 2008; Suchak and de Waal, 2012; Snippe et al., 2018). When facing threat (COVID-19), compared with high relational mobility, people may expect more reciprocity in the low relational mobility environment and thus show more helping behaviors.

## Conclusion

We found that relational mobility promoted helping before the COVID-19 pandemic but reduced helping during the pandemic. The results shed light on how ecological and socioecological contexts alter helping behaviors.

## Data availability statement

The original contributions presented in the study are included in the article/supplementary material, further inquiries can be directed to the corresponding author.

## Ethics statement

This study was approved by the Committee of Protection of Subjects at Shenzhen University. The patients/participants provided their written informed consent to participate in this study.

## Author contributions

XZhang contributed to the study conception, design, material preparation, data analysis, and writing draft. XZhao contributed to revising and writing manuscript. GL, YH, and XF performed the data collection. All authors contributed to the article and approved the submitted version.

## Funding

This study was funded by Guangdong 13th-five Philosophy and Social Science Planning Project (GD20CXL06), Shenzhen Natural Science Fund (the Stable Support Plan Progam 20200813121341001), and National Natural Science Foundation of China (31600912), Natural Science Foundation of Guangdong Province, China (No. 2022A1515011838).

## Conflict of interest

The authors declare that the research was conducted in the absence of any commercial or financial relationships that could be construed as a potential conflict of interest.

## Publisher’s note

All claims expressed in this article are solely those of the authors and do not necessarily represent those of their affiliated organizations, or those of the publisher, the editors and the reviewers. Any product that may be evaluated in this article, or claim that may be made by its manufacturer, is not guaranteed or endorsed by the publisher.

## Footnotes

^1^For most participants, an important reason of participating a psychological experiment is to obtain payment or credits, because it takes time and effort. Hence, if a person wants to participate in a stranger’s experiment for free, it can be regard as offering help.

^2^Because we made telephone calls to the participants for a short anonymous interview, we did not ask more about participants’ personal information and did not collect participants’ age. Although we did not collect participants’ age, we inferred that the participants’ age should be similar to Study 1. Because both of the two studies were sampled from the same university.

^3^We conducted a correlational analysis between interviewer’s gender (male = 1, female = 2) and participants’ helping decisions (help = 1, no help = 2), *r* = 0.02, *p* = 0.827. Hence, there was not an experimenter’s gender effect on helping.

^4^Because we made telephone calls to the participants for a short anonymous interview, we did not ask more about participants’ responses to manipulation check questions as in Study 1.
